# Impact of the COVID-19 pandemic on a clinical trial of pneumococcal vaccine scheduling (PVS) in rural Gambia

**DOI:** 10.1186/s13063-023-07298-w

**Published:** 2023-04-14

**Authors:** Ilias Hossain, Isaac Osei, Galega Lobga, Baleng M. Wutor, Yekini Olatunji, Williams Adefila, Banjo Adeshola, Yasir Isa, Cattram Nguyen, Kemo Sonko, Lamin Ceesay, Bubacarr Baldeh, Omar Barrow, Benjamin Young, Saidina Ceesay, Abdoullah Nyassi, Golam Sarwar, Ousman Barjo, Momodou M.Drammeh, Rasheed Salaudeen, Grant Mackenzie

**Affiliations:** 1 Medical Research Council Unit The Gambia at London School of Hygiene & Tropical Medicine, PO Box 273, Fajara, Banjul, The Gambia; 2 Faculty of Infectious & Tropical Diseases, London School of Hygiene & Tropical Medicine, London, UK; 3 Murdoch Children’s Research Institute, Melbourne, Australia; 4 Regional Health Directorate, Upper River Region, Ministry of Health, Basse, The Gambia; 5 Regional Health Directorate, Central River Region, Ministry of Health, Bansang, The Gambia; 6 Department of Paediatrics, University of Melbourne, Melbourne, Australia

**Keywords:** Pneumococcal vaccine, COVID-19, Pandemic, Immunisation, Clinical trial

## Abstract

The COVID-19 pandemic represents an unprecedented challenge for clinical research. The Pneumococcal Vaccine Schedules (PVS) study is a non-inferiority, interventional trial in which infants resident in 68 geographic clusters are randomised to two different schedules for pneumococcal vaccination. From September 2019 onwards, all infants resident in the study area became eligible for trial enrolment at all Expanded Programme on Immunisation (EPI) clinics in the study area. Surveillance for clinical endpoints is conducted at all 11 health facilities in the study area. PVS is conducted as a collaboration between the Medical Research Council Unit The Gambia (MRCG) at LSHTM and the Gambian Ministry of Health (MoH). The COVID-19 pandemic caused many disruptions to PVS. MRCG instructed interventional studies that participant enrolment be suspended on 26 March 2020, and a public health emergency was declared in The Gambia on 28 March 2020. Enrolment in PVS restarted on 1 July 2020 and was suspended again on 5 August 2020 after The Gambia experienced a sharp increase in COVID-19 cases in late July 2020 and restarted again on 1 September 2020. During periods of suspended enrolment of infants at EPI clinics, PVS continued safety surveillance at health facilities, albeit with disruptions. During the periods of suspended enrolment, infants who had been enrolled before 26 March 2020 continued to receive the PCV schedule to which they had been randomly allocated based on their village of residence, whereas all other infants received the standard PCV schedule. Throughout 2020 and 2021, the trial faced numerous technical and operational challenges: disruption to MoH delivery of EPI services and clinical care at health facilities; episodes of staff illness and isolation; disruption of MRCG transport, procurement, communications and human resource management; and also a range of ethical, regulatory, sponsorship, trial monitoring and financial challenges. In April 2021, a formal review concluded that the pandemic had not compromised the scientific validity of PVS and that the trial should continue as per protocol. The continuing challenges that COVID-19 poses to PVS, and other clinical trials will persist for some time.

**Supplementary Information:**

The online version contains supplementary material available at 10.1186/s13063-023-07298-w.

## Background and introduction

The Pneumococcal Vaccine Schedules (PVS) study is a parallel-group, phase IV, unmasked, non-inferiority, cluster-randomised field trial of an alternative compared to the standard schedule of pneumococcal conjugate vaccination (PCV) in rural Gambia [1, 2]. The alternative schedule includes one early dose and one booster dose scheduled at 6 weeks and 9 months (i.e. a ‘1 + 1’ schedule) while the standard schedule includes three early doses without a booster, scheduled at 6, 10 and 14 weeks (i.e. a ‘3 + 0’ schedule) of age. PVS measures the population-level effects of the schedules on endpoints of pneumococcal carriage and disease. A nested sub-study measures endpoints of pneumococcal acquisition, immunogenicity and immune response to the co-administration of PCV and yellow fever (YF) vaccine at the individual level [2]. During the 4 years of trial intervention, approximately 40,000 infants will receive the interventions, and a population of approximately 45,000 children will be under surveillance for clinical outcomes. The trial is located in the Basse and Fuladu West Health & Demographic Surveillance Systems (BHDSS and FWHDSS) in the Upper and Central River Regions (URR and CRR), respectively. PVS is conducted as a collaboration between the Medical Research Council Unit The Gambia (MRCG) at London School of Hygiene & Tropical Medicine (LSHTM) and the Gambian Ministry of Health (MoH). Vaccines are administered by MoH staff at 68 geographically distinct Reproductive Child Health (RCH)/Expanded Programme on Immunisation (EPI) clinics. Group allocation in the trial is determined by the village of residence of infants arranged in 68 geographic clusters, each assigned to one EPI clinic.

Currently, the global impact of PCV is limited by its cost. PVS will generate evidence in a typical African setting to address the global health priority to reduce the cost of PCV and so increase the global use, sustainability and coverage of PCV. Data from The Gambia will be essential to WHO considerations of revised global recommendations for the scheduling of PCV [1, 3].

Clinical trials are essential to provide evidence on the safety and efficacy of medical interventions [4]. Many disruptions to clinical trials due to the COVID-19 pandemic occurred in 2020 with ongoing effects in 2021 and 2022. Every trial in Africa has been severely impacted by the pandemic, and many have had to stop or were so severely affected that initial objectives became unattainable [5–7]. As with other countries, COVID-19 came to The Gambia in early 2020 [8, 9]. MRCG at LSHTM responded to the pandemic aiming for staff safety, ethical conduct towards trial participants, pandemic response, minimising data loss and adherence to government directions.

PVS began participant enrolment on 22 August 2019. The delivery of the trial interventions began on 2 September 2019. Surveillance for clinical endpoints began on 9 September 2019. Throughout 2020 and 2021, PVS and MRCG encountered numerous technical and operational challenges: disruption to MoH delivery of EPI services and clinical care at health facilities; episodes of staff illness and isolation; disruption of MRCG laboratory services, transport, procurement, communications and human resource management; and also a range of ethical, regulatory, sponsorship, trial monitoring and financial challenges. Here, we discuss the impact of the pandemic on PVS, which is currently ongoing.

## Enrolment

The Gambia confirmed its first COVID-19 case on 17 March 2020. MRCG instructed interventional studies to suspend participant enrolment on 26 March 2020, and all activities associated with observational studies were suspended. The Gambian government declared a State of Emergency on 27 March 2020. Interventional studies were instructed to document and discuss actions with participants, trial sponsors, funders, Data Monitoring Committees (DMC), Trial Steering Committees (TSC), Ethics Committees and funding agencies. Meetings of the PVS DMC and TSC in April 2020 concurred with the suspension of enrolment. MRCG continued to monitor the spread of COVID-19 in the country, liaise with government authorities and regularly update its guidance to clinical trials being conducted by the unit. Close consultation was maintained throughout with the MoH Regional Health Directorates in the URR and CRR as well as with each RCH/EPI team in the study area.

**Fig. 1:**
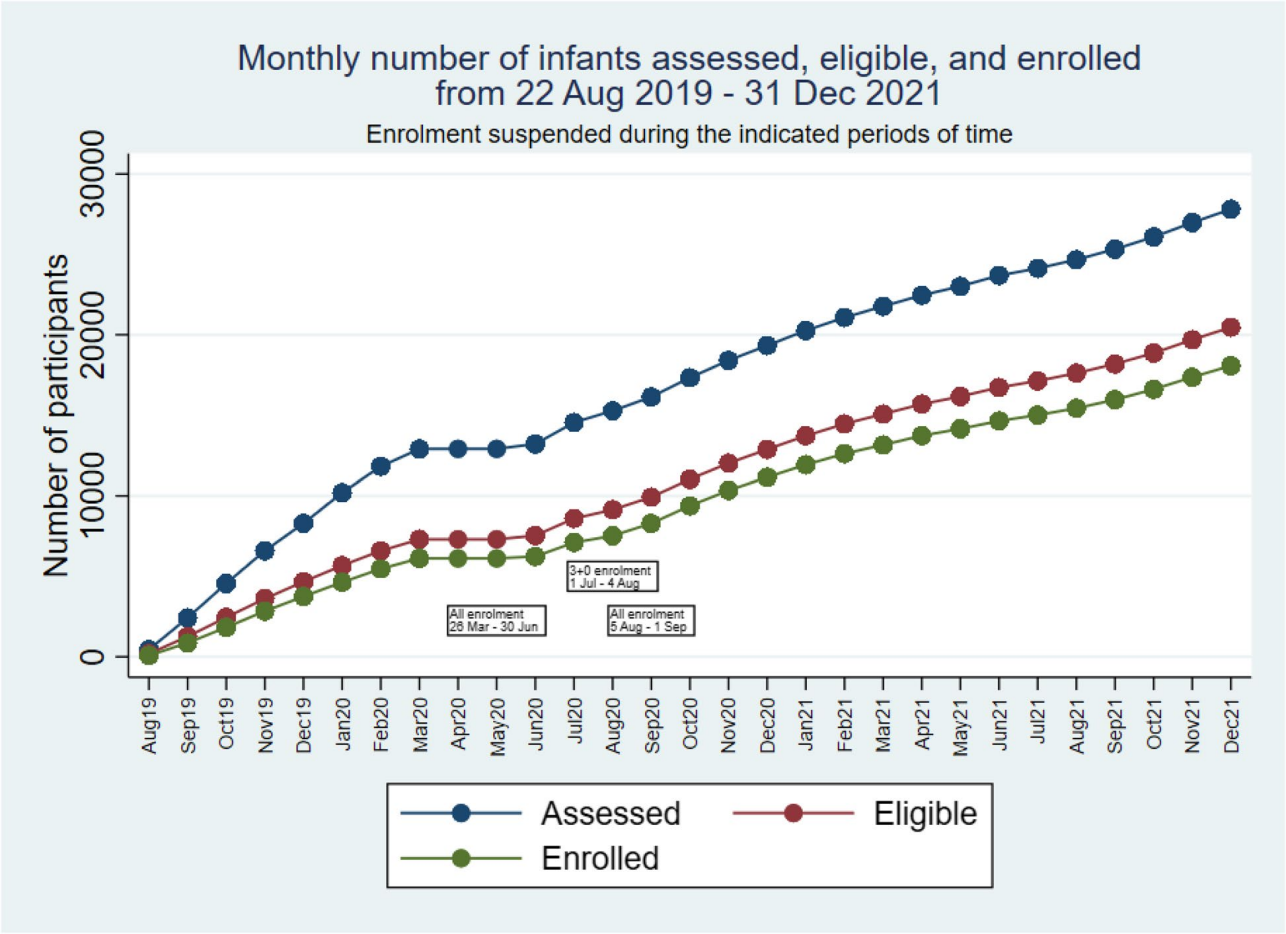
Cumulative numbers of infants assessed for eligibility and enrolled at EPI clinics

**Fig. 2:**
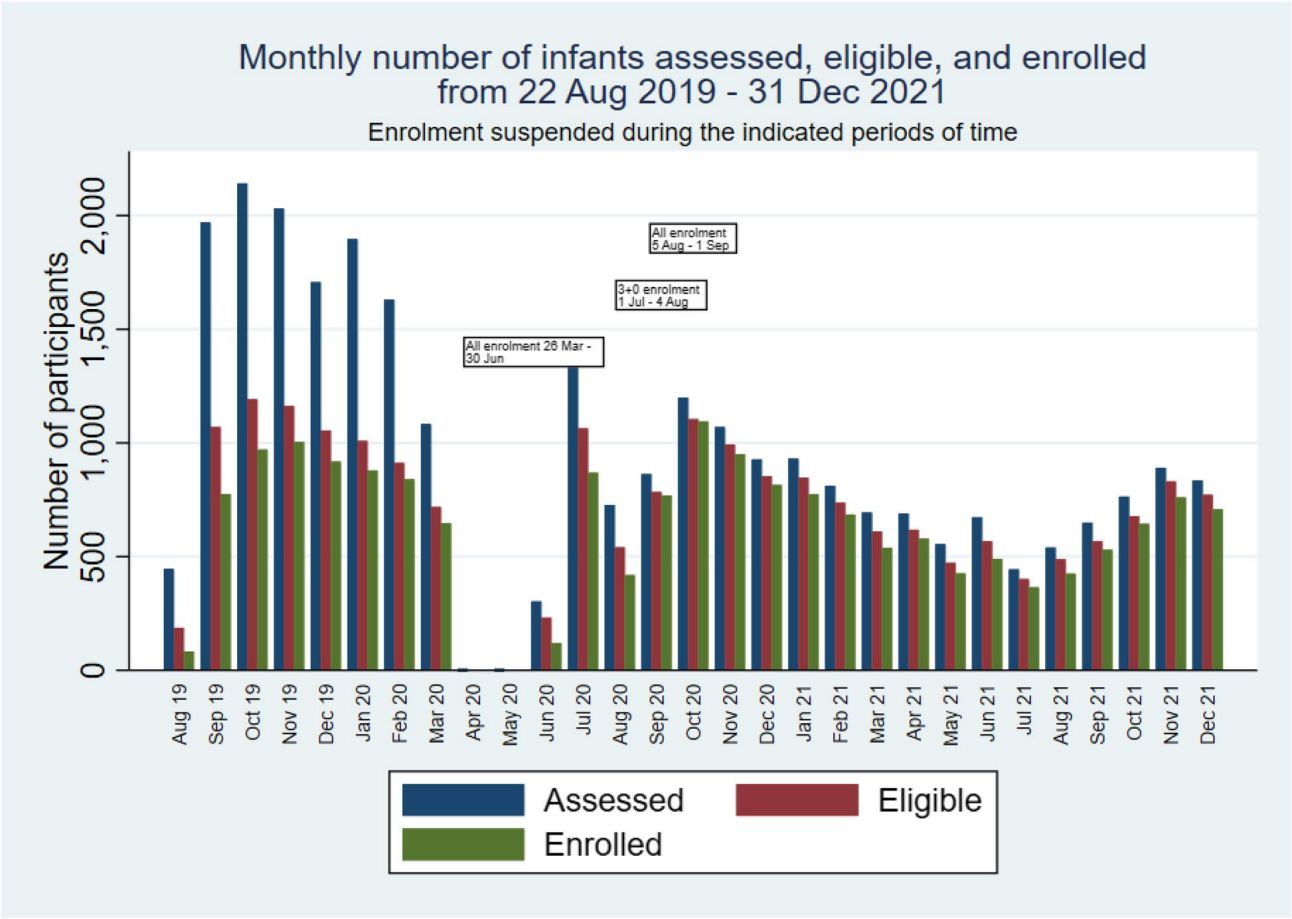
Monthly numbers of infants assessed for eligibility and enrolled at EPI clinics

PVS and RCH/EPI clinic staff collaborate to conduct PVS procedures. Field staff attend each RCH clinic (4–5 clinics held each day, Monday to Friday). PVS staff assist RCH staff to screen infants attending RCH clinics to determine whether immunisations are due. PVS staff enrol participants and prepare documentation, and EPI staff administer the vaccines. Screening, enrolment and vaccination data are recorded electronically in real time. Following the detection of the initial cases of COVID-19 and the initial suspension of enrolment, very few additional cases of COVID-19 were detected in the country in April, May and June 2020. Piloted restart of recruitment began on 1 July 2020 alongside heightened community engagement to educate participants and parents in multiple areas including, the importance of infants attending RCH/EPI clinics, measures to prevent transmission of COVID-19, the need for children to carry a health card at presentation to any health facility as well as provision of hand sanitising stations at RCH/EPI clinics along with the streamlined flow to minimise over-crowding. It was emphasised that PCV is a routine vaccine procured by the EPI and defaulters were traced to improve vaccination coverage and prevent epidemic meningitis, measles and whooping cough. Routine recruitment in villages assigned to the alternative schedule restarted on 6 July 2020 to allow a phased introduction of new procedures at RCH clinics and to focus on ‘catch-up’ enrolment in the alternative schedule group. However, The Gambia experienced a sharp increase in COVID-19 cases in late July 2020 [8], and as a result, on 5 August 2020, the unit again suspended enrolment in interventional studies. Following the establishment of additional procedures in PVS and MRCG to minimise risk in the pandemic setting, and increase awareness of the generally mild or asymptomatic clinical presentation of infection with SARS-CoV-2 in our setting [10], PVS followed unit directions and restarted participant enrolment on 1 September 2020. At this time, we restarted enrolment in both trial groups. Participant enrolment then continued without further pandemic-related interruption. Enrolment over time, along with the periods of suspension, is illustrated in Figs. 1 and 2.

Initiation of enrolment in the sub-study of pneumococcal acquisition and immunogenicity was delayed due to the COVID-19-related suspension of trial enrolment. Enrolment finally began on 14 September 2020 and was completed on 28 October 2021.

## Delivery of the intervention

During the periods of interrupted enrolment, RCH/EPI services continued. Infants enrolled before 26 March 2020 continued to receive the PCV schedule to which they were allocated in the study and which was indicated on their infant welfare card. The DMC and TSC deemed there was less risk to participants if EPI staff followed the schedule indicated on the infant welfare card rather than risk confusion by asking EPI staff to administer PCV in a schedule contrary to that recorded on the infant welfare card. EPI staff were confident to continue implementing the two different schedules as recorded on infant welfare cards. Infants presenting to EPI clinics during the periods of suspended enrolment who had not been enrolled in PVS received the standard schedule for PCV (i.e. the 3 + 0 schedule) irrespective of the group allocation of their village of residence. The sponsor and Ethics Committee concurred with this approach. This meant that unenrolled infants who were residents in villages allocated to the alternative schedule received the standard schedule during these periods. Such infants who received a full 3 + 0 schedule were classified as ‘cross-over’ from the alternative to the standard schedule group. The trial’s statistical analysis plan defines ‘cross-over’ from alternative to standard schedule related to receiving three or more doses of PCV at an age when doses will not function as a booster, as is the intention of the standard schedule. Cross-over is defined as the administration of (a) three doses of PCV with the administration of the third dose < 252 days (< 36 weeks) of age or (b) four doses with the administration of the fourth dose < 252 days (< 36 weeks) of age [3]. At the end of 2020, the cross-over of infants resident in alternative schedule villages was 4.6% (340/7463), primarily due to declined consent rather than lack of enrolment due to pandemic-related suspension.

EPI staff continued to hold all immunisation sessions and administered doses of PCV according to each participant’s allocated schedule recorded on the infant welfare card. Trial staff attending EPI clinics were reduced in number, however continuing to ensure that EPI staff issued infant welfare cards to newborns and recorded vaccination dates therein. The trial stopped recording newborn registrations and vaccination dates and ceased defaulter tracing from 26 March to 8 June 2020. Trial staff attending RCH clinics during this period assisted RCH teams with the conduct of clinics, screening infants for routine immunisation, facilitating the flow of parents and infants through the clinic and informing parents of the approach taken by the study. They also assisted in advising parents on infection prevention and control, providing infant welfare cards if not available through the EPI service, informing parents of the importance of continuing routine immunisation and coordinating the delivery of the two different schedules to enrolled participants. The RCH/EPI service in the study area experienced substantial challenges during this time and the announcement of the state of emergency, and government messaging led to concerns and fear in the public which in turn led to a general reluctance to attend public gatherings. Also, rumours of unauthorised testing of COVID-19 vaccines caused some concern in the community but were strongly refuted by government agencies. Attendance at RCH/EPI clinics was substantially reduced in the months of April, July and August, but with subsequent rapid return to normal attendance and recovery of antigen delivery to the infant population [11]. Upon resumption of trial activities at RCH/EPI clinics, field staff were at physically separate locations, with physical distance, masks and hand hygiene. Staff endeavoured to conduct consenting before the clinic started, to facilitate physical distancing. The staff washed their hands with soap and water or used alcohol-based hand sanitisers after attending to each child and parent. The study provided tap-fitted buckets to all 68 RCH/EPI clinics to facilitate hand hygiene for any person attending the clinic.

Delivery of the YF vaccine in the sub-study of PCV/YF vaccine co-administration was disrupted due to pandemic-related stock-out of the YF vaccine in some EPI clinics in September 2021. The study team arranged for the administration of the YF vaccine at alternative clinics in the study area where the vaccine was in-stock.

## Endpoint surveillance

Measurement of the primary endpoint of nasopharyngeal (NP) pneumococcal carriage in children aged 2 weeks to 59 months with clinical pneumonia at health facilities was suspended from 27 March to 13 September 2020 due to the pandemic. MRCG had instructed trials to suspend the collection of specimens unrelated to participant safety or specimens that may be associated with the transmission of respiratory viruses. The resumption of NP specimen collection following health and safety review and modification of specimen collection and laboratory procedures occurred at a convenient time given the per-protocol measurement of the pneumococcal carriage endpoint was specified in year 2 of intervention, which began on 1 October 2020. Of the 3888 children with clinical pneumonia as of 31 December 2020, NP specimens were collected from 3107 with 457 NP specimens not collected due to the pandemic-related suspension of NP specimens and 261 declined consent for NP collection.

The monitoring of safety in PVS is primarily related to clinical surveillance for invasive pneumococcal disease and radiological pneumonia among children aged < 5 years and residents in the study area. Surveillance for these clinical safety endpoints at health facilities continued throughout the pandemic interruption period although, in a scaled-back manner at the two main hospitals in Basse and Bansang from 27 March to 27 May 2020. During this period, clinical staff received regular training on infection prevention, additional personal protective equipment, hand sanitisers and training on modified study-specific procedures to reduce the risk of infection. The reduction in the intensity of surveillance was due to a unit direction instructing PVS clinical staff to prioritise support for government paediatric services. Meetings with the DMC and TSC in April, May and June 2020 recommended that clinical safety surveillance continue and resumed at full scale. The sponsor was informed of this recommendation on 20 May 2020. Clinical safety surveillance was re-established in a stepwise manner from 28 May and returned to full scale by 19 August 2020.

Clinical safety surveillance was fraught with difficulties. An interruption occurred at Bansang Hospital where our staff experienced exposure to a COVID-19 case and were in isolation from 15 July to 3 August 2020. Unfortunately, the Bansang team experienced another exposure and was isolated from 4 to 31 August 2020. We reinforced infection prevention procedures and re-structured staffing arrangements to minimise the risk of whole team exposure and total study interruption. Numerous interruptions to staffing capacity throughout the PVS team occurred due to COVID-19, from one clinical staff being unable to return from overseas for 6 months to repeated events of staff quarantine and isolation. The resumption of full safety surveillance was completed with the provision of psychological support for clinical staff and regular testing for SARS-CoV-2. The clinical staff wore an apron, surgical mask and gloves at all times. The staff wore eye protection and washed their hands after every patient encounter. The staff performed hand hygiene as often as possible or immediately after hand/skin contact with secretions. Staff kept a minimum time (max. 15 min) in contact with a patient with respiratory symptoms and instances of necessary prolonged care necessitated rotation of staff members. NP swab collection required the use of an N95 mask, goggles and an apron, before and after hand hygiene. If a patient was suspected of having COVID-19, no sample was taken by the study staff but rather the national COVID-19 team was informed to implement the appropriate protocol.

X-ray procedures for safety surveillance continued throughout the period in Basse. X-ray procedures in Bansang could not be implemented in a standardised manner due to hospital restrictions on the criteria for patients to have X-rays performed. X-ray procedures resumed a standardised approach site by site in Bansang and the two other sites performing X-rays with full activity resumed on 19 August 2020. Staff performing X-rays for patients aged < 5 years used masks, hand hygiene, gloves and physical distancing as possible. Drivers did not have direct contact with patients, and they used hand hygiene and frequent disinfection of touched surfaces. Unwell staff were advised not to present to work.

## Laboratory activities

MRCG attended a CDC-Africa regional workshop on the diagnosis and laboratory testing for novel coronavirus disease in February 2020 in Dakar, Senegal. Thereafter, unit laboratory protocols for biosafety, specimen collection, processing and testing for suspected coronavirus disease-infected samples used WHO guidelines [12, 13]. The same procedures were adapted for use in PVS. The culture of NP specimens in the Basse laboratory safety cabinet required the use of a gown, gloves, goggles and mask.

## Data management

Data management including new data collection, weekly data synchronisation, data cleaning, generating queries, validation checking, quality control and quality assurance continued amidst substantial challenges throughout the suspension period.

## Demographic surveillance

Demographic surveillance to maintain the population list and detect births and deaths was suspended from 26 March to 1 June 2020 and then suspended again between 5 August and 1 September 2020. Field staff restarted visiting HDSS village reporters to continue the recording of birth and death events. Field staff maintained weekly communications with village health workers and other community representatives. Events not recorded during the periods of suspension were detected and recorded when demographic surveillance resumed.

## Risk management

We conducted two phases of formal risk assessments of clinical surveillance, field procedures at RCH/EPI clinics and demographic surveillance, which were reviewed by MRCG health and safety and unit leadership and activities resumed only after their approval. Management of risk comprised a wide range of measures, including training of all staff in general and specific aspects of infection prevention, provision and supervision of personal protective equipment for all staff, physical distancing, specimen handling, psychological support, health check-ups and periodic reassessment. The trial provided additional masks, hand gel, crowd control and handwashing stations at RCH/EPI clinics. The study identified 16 RCH clinic sites with inadequate facilities to support infection prevention and invested to improve the infrastructure at those sites.

## MRCG unit and trial adjustments

All staff received a risk allowance for 5 months. MRCG experienced severe difficulties, which are ongoing, in the procurement of consumables. Airfreight was used much more often to ensure timely procurement. To overcome the failure of suppliers to provide sheep blood for NP specimen culture, we procured two sheep from which we obtained blood for microbiology requirements. All these issues led to increases in the rate of expenditure in the trial.

## Review of scientific validity

Although the difference between the number of infants eligible and enrolled increased after the period of pandemic interruption (Fig. 1), primarily due to suspensions of enrolment, the difference was less than expected and not extreme. The slight trend of an increasing gap between numbers eligible and enrolled in 2021 suggest an ongoing dynamic that is unrelated to the pandemic but not threatening the validity of the trial. As of 31 December 2021, the percentage of eligible infants enrolled was 91.3% (18,747/20,544).

The rate of declined consent did not change in the period before compared to during the pandemic. As of 31 December 2021, the overall decline of consent was 4.2% (812/19,559), 3.4% (370/11013) in the alternative schedule group and 5.2% (442/8546) in the standard schedule group. Approximately 40% of declined consents were located in two RCH/EPI clinic sites. Community meetings have been held in these two locations to explore the concerns leading to declined consent.

The difference in numbers enrolled in the two groups at the end of 2021; *n* = 10,643 (alternative schedule) and *n* = 8104 (standard schedule), relates to per-protocol enrolment between 22 August 2019 and 7 February 2020 with increased emphasis on enrolment of infants up to 9 months of age in the alternative schedule group, while in the standard schedule group enrolment criteria included infants who had not yet received the 3rd dose of PCV or were less than 6 months of age [1]. Baseline characteristics were similar in infants aged 6–8 months and resident in standard schedule villages and not enrolled compared to those residents in alternative schedule villages and enrolled. In addition, there was focused catch-up enrolment in the alternative schedule group only from 1 July to 4 August 2020 following the period of pandemic-related suspension of enrolment.

The characteristics of infants enrolled at EPI clinics until 31 December 2021 appear similar in the two groups (Table 1).

**Table 1:** Characteristics of infants enrolled at EPI clinics

Characteristic	Schedule group			
	Alternative, *n* = 10,643		Standard, *n* = 8104	
	Median	Interquartile range	Median	Interquartile range
Age of mother (years)	27	21–33	27	22–33
Age at 1st dose PCV (weeks)	10	8–12	10	8–12
Age at 3rd dose pentavalent (weeks)	20	18–24	21	18–25
Age at 1st dose measles (weeks)	42	41–45	42	41–45

Infants resident in alternative schedule villages who were not enrolled during the COVID-19 interruption and who may have received the standard schedule will constitute a ‘cross-over’ between the two groups. Cross-over will reduce any difference in effect between the two groups with a potential bias towards the null. As of 31 December 2020, the proportion of all alternative schedule village resident infants registered at EPI clinics who were cross-overs was 4.6% (340/7463). Given that cross-over from alternative to standard schedule is primarily related to the decline of consent, i.e. currently 3.4%, we project that in year 4 of the trial the proportion will be between 3 and 4%. We statistically evaluated the potential impact of cross-over bias using simulations based on the assumptions used in the original sample size calculations. The simulation methods were as follows:

- Simulate 68 clusters of 60 participants
- Assume no cross-over in the standard schedule arm
- Simulate cluster-randomised treatment as ${A}_{i}\sim \mathrm{Bern}(0.5)$
- Simulate a binary individual-level indicator for cross-over. Let ${C}_{ij}=1$ for compliers, 0 otherwise.
- Individual-level treatment received is derived as:
  $${D}_{ij}={C}_{ij}{A}_{j}$$

so that individuals in the standard schedule arm will always receive 3 + 0, but those in the alternative schedule arm can switch to 3 + 0.

The following model was used to simulate the data:

$${\mathrm{logit}(\pi }_{ij})=\mu +{\beta }_{C}{C}_{ij}+{\beta }_{CA}{C}_{ij}{A}_{i}+{b}_{i}$$

where

 ${\pi }_{ij}$ = ${\mathrm{Pr}(y}_{ij}=1)$, where ${y}_{ij}$ is vaccine-type carriage status for the *j*th child in the *i*th cluster

$\mu$ = log-odds of carriage prevalence in the standard schedule arm when the random effect is zero

${\beta }_{C}$ = direct effect of compliance

${\beta }_{CA}$ = log of the odds ratio comparing receipt of the 1 + 1 schedule compared to the 3 + 0 schedule

${b}_{i}$ = cluster-level random effect. Assume it is normally distributed with mean zero and standard deviation ${\sigma }_{c}$, i.e. ${b}_{i}\sim N(0,{\sigma }_{c})$.

We simulated 2000 datasets with a random selection of alternative schedule participants to cross-over. The vaccine-type pneumococcal carriage outcome was simulated using a multi-level logistic model, assuming an intra-cluster correlation of 0.02. The following scenarios were explored:

- Prevalence of vaccine-type carriage in the standard schedule arm = 13%, 15%
- Odds ratio comparing the alternative and standard schedule arms = 1, 1.1, 1.2 (corresponding to “true” values of the prevalence ratio of 1, 1.09, 1.17 for 13% prevalence, and 1, 1.08 and 1.17 for 15% prevalence)
- Percentage of cross-over = 3%, 4%, 6%, 8%, 10%

Bias was calculated as the difference between the average estimate of the prevalence ratio minus the true value. Power was calculated as the proportion of simulations where the upper limit of the 95% confidence interval of the pre-defined non-inferiority margin prevalence ratio was below 1.38 [1, 3]. Statistical coverage was calculated as the proportion of simulations where the true value of the prevalence ratio was within the simulated 95% confidence interval.

The estimated study power in 2000 simulations for the scenario of 13% vaccine-type prevalence in each arm and 6% cross-over was 91.2%. Estimated levels of bias in the prevalence ratio with different degrees of cross-over were quite limited, around 0.005 for all levels of cross-over. Study power with different degrees of cross-over was influenced by only a minimal degree. Study power varied when the true prevalence of vaccine-type carriage varied in the two groups. When true vaccine-type prevalence was 13% in each group, study power was around 91% to demonstrate non-inferiority. However, when true vaccine-type prevalence was 13% in the standard schedule group and 14% in the alternative schedule group study power was around 73% to demonstrate non-inferiority.

**Fig. 3:**
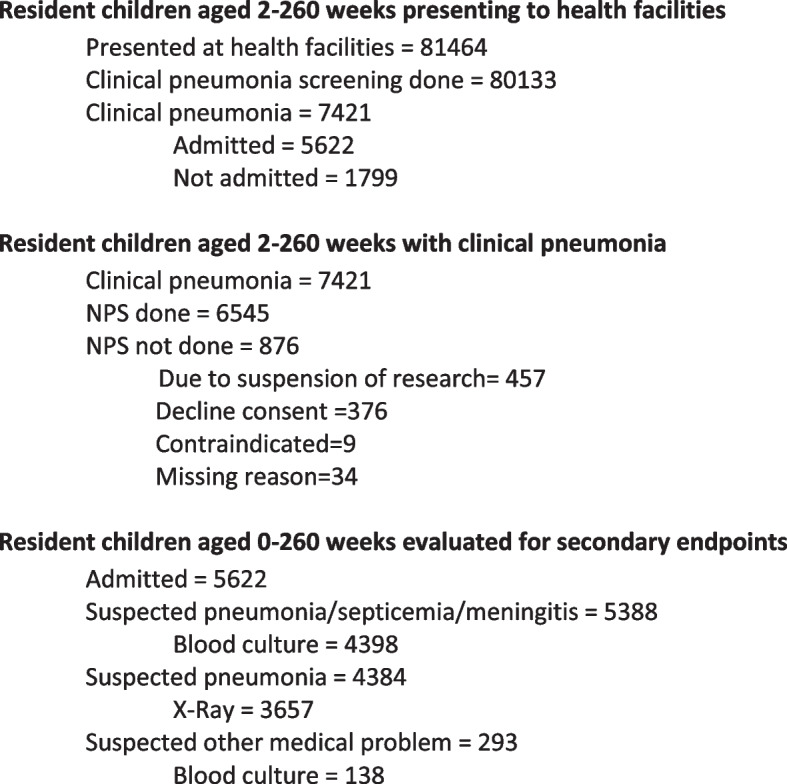
Profile of clinical surveillance of children presenting to health facilities until 31 December 2021

Measurement of primary and secondary endpoints involves patient investigation at health facilities. Figure 3 shows patient presentations and investigations until 31 December 2021. The numbers of presentations and investigations are lower than expected due to the pandemic’s negative effect on healthcare seeking and access to care. Trial sample size calculations for the primary endpoint of vaccine-type pneumococcal carriage among children aged 2–260 weeks with clinical pneumonia in year 4 require measurement in 4080 patients. We had measured this endpoint in 7421 patients by 31 December 2021. We expect that without pandemic-related hindrances to health care seeking and access to care in year 4 patient numbers will increase to meet the required sample size. We also plan a protocol amendment to allow an extension of the year 4 period by 1 or 2 months if insufficient numbers of patients present within the 12-month period, and so ensure the required sample size.

## Conclusion

The pandemic affected all facets of PVS as with many other trials over the world. Adequate measures were put in place to mitigate the impact on study objectives and also to protect participants and staff from infection. There was minimal cross-over, and the scientific validity of the study was maintained. The pandemic is not over and in collaboration with the MoH; continuing efforts will be made to ensure the smooth operation of the trial while adhering to the guidelines provided by MoH and MRCG. The EPI clinics have streamlined procedures to maintain physical distance and infection control. The MoH is committed to the continued implementation of EPI services. The project collaborated with the MoH to support regional health authorities to mitigate the impact of COVID-19 at EPI clinics and health facilities. The project is now operating at full scale and has completed delivery of the intervention for 16 months of the per-protocol 48-month intervention period.

## Supplementary Information

**Additional file 1: Figure 4.** Study sheep providing blood for the culture of microbiology specimens. **Figure 5.** COVID-19 cases in The Gambia

## Data Availability

Data will be made available on request.

## References

[1] Mackenzie GA, Osei I, Salaudeen R, Secka O, D’Alessandro U, Clarke E (2022). Pneumococcal conjugate vaccination schedules in infants-acquisition, immunogenicity, and pneumococcal conjugate and yellow fever vaccine co-administration study. Trials.

[2] Mackenzie GA, Osei I, Salaudeen R, Hossain I, Young B, Secka O (2022). A cluster-randomised, non-inferiority trial of the impact of a two-dose compared to three-dose schedule of pneumococcal conjugate vaccination in rural Gambia: the PVS trial. Trials.

[3] Mackenzie GA, Palmu AA, Jokinen J, Osei I, Flasche S, Greenwood B, Mulholland K, Nguyen C (2022). Pneumococcal vaccine schedules (PVS) study: a cluster-randomised, non-inferiority trial of an alternative versus standard schedule for pneumococcal conjugate vaccination-statistical analysis plan. Trials..

[4] Guenter D, Esparza J, Macklin R (2000). Ethical considerations in international HIV vaccine trials: summary of a consultative process conducted by the Joint United Nations Programme on HIV/AIDS (UNAIDS). BMJ J Med Ethics.

[5] Chan CHS, Tan EK (2020). Safeguarding non-COVID-19 research: looking up from ground zero. Arch Med Res.

[6] Yanow SK, Good MF (2020). Nonessential research in the new normal: the impact of COVID-19. Am J Trop Med Hyg.

[7] Johns Hopkins University. Coronavirus Resource Center. 2022. https://coronavirus.jhu.edu/. Accessed 29 Sep 2022.

[8] Ministry of health the Gambia. Gambian government COVID-19 updates. 2022. https://www.moh.gov.gm/covid-19-report/.

[9] Worldometer. World population by region. Worldometer. 2022. https://www.worldometers.info/world-population/#region.

[10] Diallo BA, Usuf E, Ceesay O, D’Alessandro U, Roca A, Martinez-Alvarez M (2022). Clinical research on COVID-19: perceptions and barriers to participation in the Gambia. BMJ Glob Heal.

[11] Osei I, Sarwar G, Hossain I, Sonko K, Ceesay L, Baldeh B (2022). Attendance and vaccination at immunization clinics in rural Gambia before and during the COVID-19 pandemic. Vaccine.

[12] World Health Organization. Laboratory biosafety guidance related to coronavirus disease (COVID-19). 2020. https://www.who.int/publications/i/item/laboratory-biosafety-guidance-related-to-coronavirus-disease-(covid-19).

[13] World Health Organization. Laboratory testing for coronavirus disease 2019 (COVID-19) in suspected human cases. 2020. https://apps.who.int/iris/handle/10665/331329.

