# Supplementary material for: Impact of the COVID-19 pandemic on a clinical trial of pneumococcal vaccine scheduling (PVS) in rural Gambia
Source: Trials. 2023 Apr 14;24:271. doi: 10.1186/s13063-023-07298-w (PMC10101732; doi:10.1186/s13063-023-07298-w)
Supplement: Supplementary file 1 — Additional file 1: Figure 4. Study sheep providing blood for the culture of microbiology specimens. Figure 5. COVID-19 cases in The Gambia [file 13063_2023_7298_MOESM1_ESM.docx]

**Additional file 1: Supplementary materials**

**Figure 4. Study sheep providing blood for the culture of microbiology specimens**


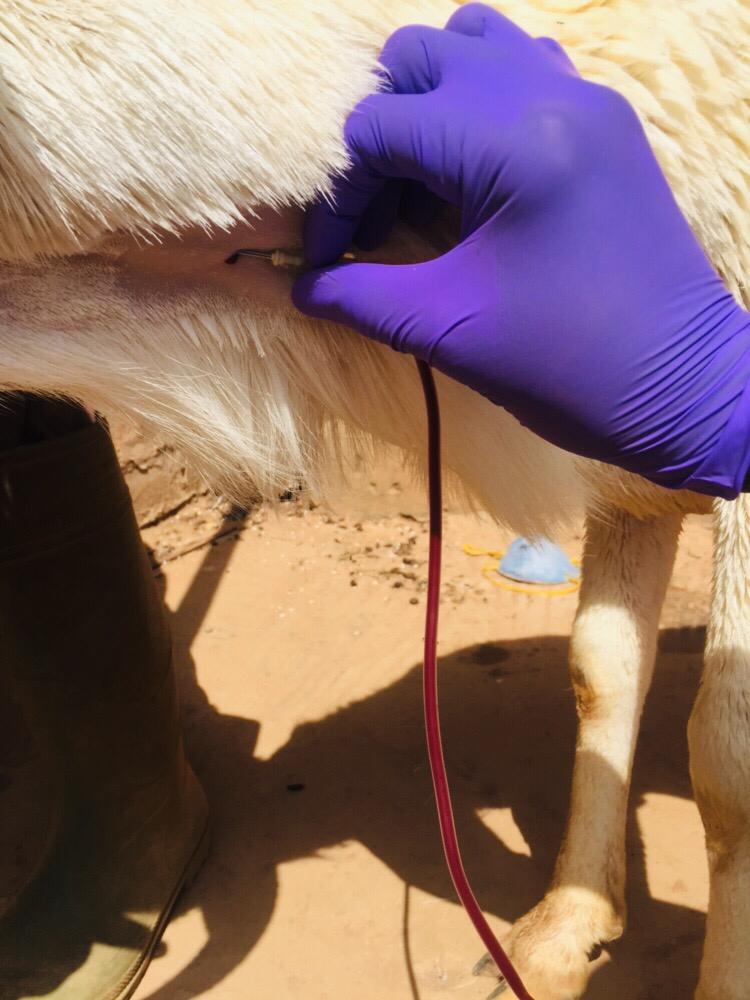
**
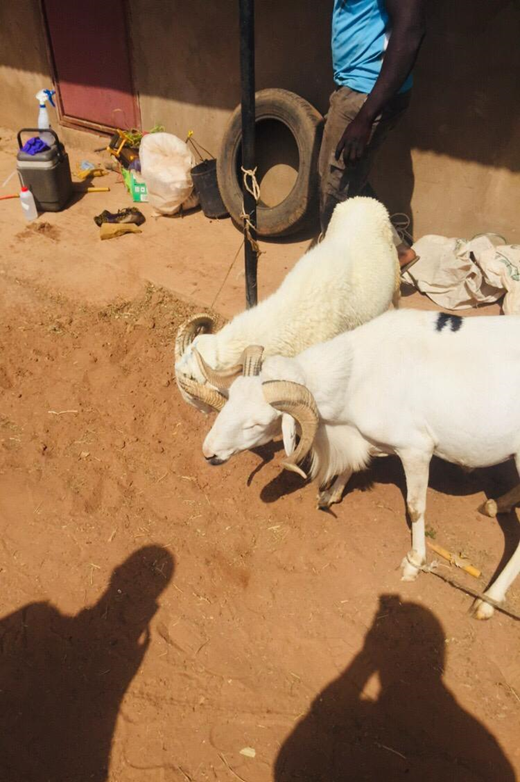
**

**Figure 5. COVID-19 cases in The Gambia**

Note: Case numbers have been very low since mid-September and less than 1% of the country’s cases have occurred in the study area.
